# Reciprocal Interactions Between Gut Microbiota and Host Social Behavior

**DOI:** 10.3389/fnint.2018.00021

**Published:** 2018-06-12

**Authors:** Emmanuelle Münger, Augusto J. Montiel-Castro, Wolfgang Langhans, Gustavo Pacheco-López

**Affiliations:** ^1^Department of Environmental Systems Science, Swiss Federal Institute of Technology (ETH) Zurich, Zurich, Switzerland; ^2^Health Sciences Department, Metropolitan Autonomous University (UAM), Lerma, Mexico; ^3^Department of Health Sciences and Technology, ETH Zurich, Zurich, Switzerland

**Keywords:** holobiont, sociality, behavioral immune system, social structure, microbiota-gut-brain axis

## Abstract

Animals harbor an extensive, dynamic microbial ecosystem in their gut. Gut microbiota (GM) supposedly modulate various host functions including fecundity, metabolism, immunity, cognition and behavior. Starting by analyzing the concept of the holobiont as a unit of selection, we highlight recent findings suggesting an intimate link between GM and animal social behavior. We consider two reciprocal emerging themes: (i) that GM influence host social behavior; and (ii) that social behavior and social structure shape the composition of the GM across individuals. We propose that, throughout a long history of coevolution, GM may have become involved in the modulation of their host’s sociality to foster their own transmission, while in turn social organization may have fine-tuned the transmission of beneficial endosymbionts and prevented pathogen infection. We suggest that investigating these reciprocal interactions can advance our understanding of sociality, from healthy and impaired social cognition to the evolution of specific social behaviors and societal structure.

## Introduction

Animals harbor a diverse community of microbes, in their gut and in almost any other site, both on and within, their bodies. Host and gut microbiota (GM) interact symbiotically (Sommer and Bäckhed, 2013). The GM contribute to host health and fitness, playing a major role in diverse host functions including development, fecundity, metabolism and immunity; for the microbes, animal intestines are a favorable niche (Shapira, 2016). By removing the need for culturing microorganisms for identification, next-generation sequencing methods have massively advanced the characterization of the human GM and are continuously expanding our knowledge about endosymbiotic microbes. We have just recently grasped that the human GM comprise hundreds of microbial species, with *Firmicutes* and *Bacteroidetes* as the dominant phyla (Lozupone et al., 2012), and that microbial life may thus have roles in multiple physiological processes including those related to mental health (Cryan and Dinan, 2012). This has opened the possibility of applying a “gestalt perspective” allowing us to understand physiological, behavioral and cognitive processes as part of an integrated whole (Koffka, 2013). Recent technological developments actually allow for the integration of data from various sources such as the genome, transcriptome, proteome, epigenome and microbiome in what has been termed “Gestaltomics”, as a useful approach to the understanding of psychiatric disorders at different levels of organization (Gutierrez Najera et al., 2017).

**Figure 1:**
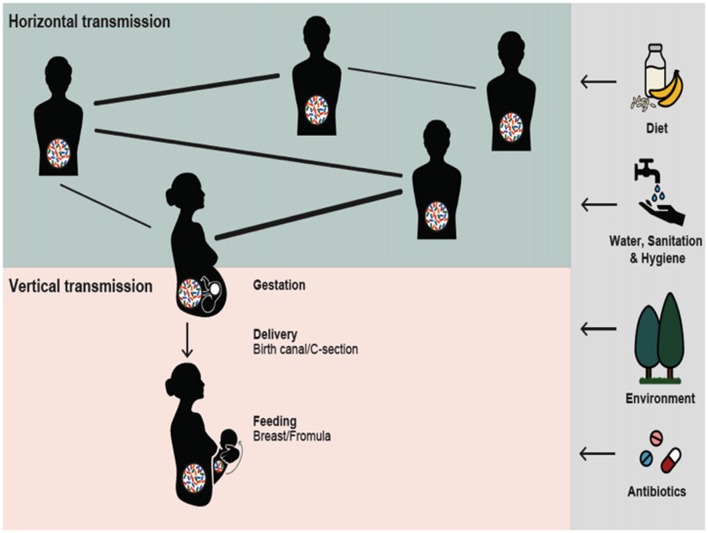
Reciprocal interactions between Gut microbiota (GM) and social structure illustrated for humans. Social interactions may allow for the horizontal GM transmission, presumably in direct relation to the strength of the social bonds (bold lines). Mothers can transmit their microbes vertically to the next generation. In reciprocity, gestation and infancy could be a critical period for the GM to influence infant brain development and future sociability. The nature and quantity of horizontally and vertically transmitted microbes may be influenced by external factors including diet, water, sanitation and hygiene, environment and antibiotic usage; vertical transmission is also influenced by the mode of delivery and method of feeding.

Aware of the perils of our inference of adaptive significance from proximate control of behavior (Dewsbury, 1999), our review proceeds as follows: in view of the accumulating evidence linking biological processes between micro- and macroorganisms, first, we suggest that the use of the concept of a holobiont as a unit of selection should be applied to the conglomerate of organisms involved in such relationship. Second, we describe the neurobiology of social behavior highlighting the possible pathways through which microbiota and, particularly, GM may affect social behavior, including macroorganisms’ development. Even under the light of recent and excellent reviews on the topic (Ezenwa et al., 2012; Archie and Tung, 2015), knowledge of the mechanisms by which microbiota in different sites of a host’s body influence behavior is still lacking. In contrast, there are at least three purported pathways suggesting how the GM interact with an individual’s central nervous system (CNS), modifying behavior in general, and social interactions in particular (Sampson and Mazmanian, 2015). For this reason, we focus our review on GM and expand this perspective by describing three areas where social behavior can in turn influence individual microbial profiles: (1) due to stressing events in hosts’ social life; (2) because of differences between solitary and social life; and (3) due to social structure. Figure 1 introduces to key elements of these interactions.

## Holobionts: “Interactors” in A Social World

Hosts’ survival and reproductive success may be at least partially dependent on the presence, characteristics and functionality of microbiota (Lombardo, 2008). Mounting evidence has even suggested the use of the concept of a “holobiont”: the individual host and its microbial communities including facultative symbionts with varied and interwoven associations (Theis et al., 2016). Moreover, the macroorganism’s genome, that of its organelles, and its microbiome, supposedly forms an aggregate known as the “hologenome” (Brucker and Bordenstein, 2013). The nascent holobiont theory suggests that: (i) all macroorganisms harbor microorganisms, serving the latter as nutrient-rich environments where to thrive; (ii) the fitness of the holobiont and its symbionts is interdependent; (iii) the hologenome can change due to variations in either the host’s genome or the microbiome; and (iv) modifications are transmissible across generations and may thus influence the holobiont’s evolution (Rosenberg and Zilber-Rosenberg, 2013b). If the symbiotic relationship of host and symbionts rather than just the macroorganism’s phenotype is selected, then the holobiont should be regarded as the relevant unit of selection (Feldhaar, 2011). In fact, there is evidence suggesting that different and characteristic communities of GM may have coevolved with carnivores, herbivores and omnivorous animals (Ley et al., 2008), or that speciation seems to have a correlate in the acquisition of specific GM (Kwong et al., 2017). Indeed, whether the holobiont actually constitutes a unit of selection is still debatable, perhaps because the term “unit of selection” itself can refer to different entities. On one hand, a holobiont may act as a unit of selection when understood as a “replicator”: an entity that through direct reproduction transmits its structure (Hull, 1980). In this sense, evidence suggests that microbiota can be accurately transmitted between holobiont generations (Rosenberg and Zilber-Rosenberg, 2013a), and that processes providing the necessary variation for selection are present in microorganisms. For instance, through modifications in the relative abundances (i.e., microbial amplification) of different microorganisms, the acquisition of genetically diverse strains from the environment, and horizontal or lateral gene transfer (Lloyd, 2017). On the other hand, a unit of selection can also be understood as an “interactor”: a unit or cohesive whole that, through direct contact with its environment (i.e., subject to selection), achieves differential replication (Hull, 1980). Including various species and populations with diverse genomes, microbial communities face both within-group (e.g., for resources) and between-group competition. Because the microbiome and the host’s genome are selected in parallel, the holobiont can be interpreted as both an individual and an interactor (Lloyd, 2017). This suggests that to grow and reproduce across different individuals and time, microbial communities should not reproduce at the (total) expense of their hosts, but that, instead, they should have mechanisms to expedite their transmission to another organism, promoting their host’s survival, reproductive success, or both (Fisher et al., 2017). Interestingly, Liao et al. (2016) recently discussed the adaptive value of diet-induced thermogenesis, proposing that it results from the coevolution of host and GM (especially *Firmicutes*) that ferment ingested food and proliferate, causing periodic, vagus nerve-mediated increases in host thermogenesis aimed at curtailing the microbial expansion. The “insurance policy hypothesis” proposes that biodiversity insures ecosystems against declines in their functioning because many species provide greater guarantees that some will maintain functioning even if others fail (Naeem and Li, 1997). Thus, because the microbial component in the holobiont can vary faster than that of the host, microbial diversity may enhance the holobiont’s adaptation when selection occurs under fluctuating environmental conditions (Lloyd, 2017). Discussing the role of the holobiont as an interactor also allows for identifying the basis of a reciprocal relationship between the GM and social behavior. GM influence several aspects of the host’s physiology. By contributing to inter-individual sociality (which can itself improve individual’s health (Nunn et al., 2015), reproductive success (Cheney et al., 2016) and general welfare (Kleinhappel et al., 2016)), GM contributes to holobiont’s survival and reproduction. This in turn may promote the transmission of GM between individual hosts while enhancing their survival.

## The Neurobiology of Social Behavior

A comprehensive understanding of the fundamental mechanisms mediating social behavior, and of how these have arisen over the course of evolution, is still very limited. However, two neural circuits that evaluate stimulus salience and/or regulate social behavior seem to be at the basis of social decision-making in vertebrates, and have recently been found to be remarkably conserved across taxa (O’Connell and Hofmann, 2011): (i) the social behavior network; and (ii) the mesolimbic reward system. The social behavior network consists of a collection of midbrain, hypothalamic and basal forebrain nuclei, that together with sex steroids and neuropeptides regulates social behavior, including reproduction, parental care and aggression. The mesolimbic reward system has a central role in connecting the dopaminergic ventral tegmental area and the nucleus accumbens, controlling stimulus’ salience mainly via dopaminergic signaling. Together, these two brain circuits constitute a larger integrated social decision making network that regulates adaptive behavior (O’Connell and Hofmann, 2011).

Beyond the deep homologies in the biological basis of social behavior, group living requires a specific set of cognitive functions. To maintain group cohesion, individuals living in social groups must be able to meet their own requirements, as well as to coordinate their behavior with other individuals in the group (Dunbar and Shultz, 2007). As such, social recognition, the ability to distinguish conspecifics’ hierarchical status, reproductive state, genetic relatedness (kin recognition), individual identity, or emotions, is crucial for intense sociality (Choleris et al., 2009). Distinct approaches have been adopted when accounting for the brain basis of complex social behavior. One of these suggests that some brain areas have a uniquely social function, whereas another purports that an aggregation of simple functions is at the basis of complex social behavior (Behrens et al., 2009). The set of areas known to be involved in the processing of social information include the anterior cingulate cortex gyrus, the dorsomedial prefrontal cortex, the tempoparietal junction and the superior temporal sulcus (Behrens et al., 2009). For instance, reward-guided behavior is known to depend on brain structures such as the orbitofrontal cortex and the amygdala, and these structures may also be involved in social perception, contributing to social decision-making (Behrens et al., 2009). In addition, recent studies suggest that the hippocampal activation of oxytocin receptors may underlie a capacity for discriminating social vs. non-social information (Raam et al., 2017). In social mammals, brain areas such as the amygdala and the prefrontal cortex have undergone important changes during evolution (Hrvoj-Mihic et al., 2013), and are critically involved in social cognition. The prefrontal cortex plays an essential role in affective processing and is, along with the amygdala, involved in emotional regulation (Davidson, 2002). In humans, the prefrontal cortex is involved in social cognition and in moral judgment processing; it is important for the modulation of urges, an essential process for an individual to fit in societies with social norms (Forbes and Grafman, 2010).

The recognition that dealing with social life in large groups presumably requires more “cognitive power” lead Dunbar (Dunbar, 1992) to examine mammals’ brain size and neocortex size in relation to social group size (i.e., as a proxy for group’s social complexity). His results suggest that species living in larger groups have larger brains (Dunbar, 2009). Using the relationship between social group size and neocortex size (calculated using the volume of fossils’ crania) allowed him to model the likely group size of several members of the hominid lineage as well as that of modern humans (Shultz et al., 2012). Concurrently, evidence suggests that multiple lineages from the human GM evolved by co-speciation with the hominid host lineage (Moeller et al., 2016a). Moreover, Stilling et al. (2014) propose that modifications of the microbiome, together with epigenetic changes and RNA-based regulation of gene expression, could have combined to promote the rapid evolution of the mammalian brain.

Oxytocin and arginine vasopressin are the major neuropeptides involved in the networks regulating social cognition and behavior (Donaldson and Young, 2008). These neurotransmitters are primarily synthesized in the supraoptic and paraventricular nuclei of the hypothalamus (Choleris et al., 2009) and are released in several brain areas or secreted by the posterior pituitary into the bloodstream to act as neurohormones (Gimpl and Fahrenholz, 2001). Oxytocin supposedly allows animals to overcome their natural avoidance of inter-individual interactions, thereby facilitating prosocial behavior (Heinrichs and Domes, 2008). It is also critically involved in both mother-infant bonding and pair bonding (Donaldson and Young, 2008). Conversely, arginine vasopressin typically mediates behaviors such as aggressiveness and territoriality, but also pair bonding and parental care (Penders et al., 2006). In humans, peripheral oxytocin levels are positively correlated with trust and trustworthiness (Zak et al., 2005) and warm physical contact with a partner (Grewen et al., 2005). In contrast, patients with autism spectrum disorder (ASD) or with schizophrenia, mental disorders associated with social deficits, have reduced peripheral levels of oxytocin (Green et al., 2001; Kéri et al., 2009).

## GM Influence on Development and Social Behavior

In humans, GM establishment during early life occurs primarily through vertical transmission from mother to offspring (Nuriel-Ohayon et al., 2016). Recent studies defy the long-held dogma that the intrauterine environment is sterile and newborns are germ-free, suggesting that microbiota is transferred from mother to fetus (Reviewed in Perez-Muñoz et al., 2017). Nonetheless, these discoveries have been strongly contested, and the “sterile womb” paradigm, proposing that the sterile fetus first acquires an early microbiome during and after birth, prevails (Perez-Muñoz et al., 2017). All the same, the mother’s GM, as well as vaginal microbiota, are known to be dramatically remodeled during pregnancy (Nuriel-Ohayon et al., 2016), supporting the suggestion that exposure to microbial metabolites and compounds originating from the maternal gut play an important role in offspring’s development (Gomez de Agüero et al., 2016; Perez-Muñoz et al., 2017). During vaginal delivery, microbiota from the maternal vagina and gut inoculate the newborn’s GM (Penders et al., 2006; Dominguez-Bello et al., 2010). In contrast, the GM of newborns delivered by C-section rather resemble maternal skin and oral microbiota (Penders et al., 2006; Dominguez-Bello et al., 2010). During infancy, the GM increase their complexity via eating and uptake of microbes from the environment (Lozupone et al., 2012). Thus, the specific GM of early life converge toward adult-like GM around the age of three (Yatsunenko et al., 2012).

Perinatal exposure to the maternal GM apparently plays an important role in the establishment of a “pioneer” infant GM, with major implications for infant brain development (Degroote et al., 2016). Human cohort studies reported an increased risk of autism spectrum disorder (ASD) in infants from obese mothers (Connolly et al., 2016) and from mothers who received antibiotic treatment during pregnancy (Atladóttir et al., 2012).

Studies in rodent models consistently demonstrated that offspring exposed prenatally to maternal high fat diet or antibiotics showed impaired sociality (Buffington et al., 2016). Buffington et al. (2016) also showed that the social impairment was mediated by mouse-pup GM, which differed between offspring from mothers fed a high fat diet and offspring from mothers fed a regular diet. Transfer of GM of offspring from mothers fed a regular diet to offspring from mothers fed a high fat diet restored normal social behavior at weaning (4 weeks), but not in adulthood (8 weeks). Furthermore, offspring from mothers fed a HFD had reduced hypothalamic oxytocin levels; treatment of these offspring with *Lactobacillus reuteri*, the most drastically reduced strain of their GM, increased hypothalamic oxytocin levels and normalized their social deficit. This suggests that *L. reuteri* improves social behavior by promoting oxytocin-mediated functions (Buffington et al., 2016). *L. reuteri* treatment also improved wound healing, supposedly by a vagally-mediated increase of oxytocin (Poutahidis et al., 2013). Recent findings from the same group suggest that *L. reuteri* viability is not essential for the regulation of oxytocin; thus, a peptide or metabolite produced by these bacteria may be sufficient (Varian et al., 2017). Degroote et al. (2016) observed that Wistar rat offspring exposed periconceptionally to antibiotics spent 50% less time in social interactions than controls. Leclercq et al. (2017) found that a low dose of penicillin administrated to mice dams from the last week of pregnancy to weaning of the pups (perinatally), reduced sociality and preference for social novelty in the offspring. Some males exposed perinatally to antibiotics exhibited a surprising aggressive behavior when physically stressed by an unfamiliar male aggressor (defeat paradigm), different from control mice, who exhibited a submissive posture (Leclercq et al., 2017). Perinatally antibiotics-treated male and female mice featured a substantially increased expression of vasopressin receptor 1b (Leclercq et al., 2017), known to be involved in social and aggressive behaviors (Wersinger et al., 2002), in the frontal cortex. Interestingly, concurrent supplementation with the probiotic strain *Lactobacillus rhamnosus* (JB-1), which supposedly regulates emotional behavior in healthy mice via the vagus nerve (Bravo et al., 2011), counteracted some of the antibiotic treatment effects. JB-1 supplementation concurrently to antibiotic treatment in fact prevented the decrease in sociality and social novelty preference in offspring, i.e., there was no significant difference between offspring of control or antibiotic treated dams in the defeat paradigm. The GM of offspring perinatally exposed to antibiotic or antibiotic + JB-1 were largely altered and clustered separately from the control group. Another recent study also demonstrated that JB-1 treatment decreased stress-induced anxiety-like behavior and prevented deficits in social interaction (Bharwani et al., 2017). These findings warrant further investigation of the influences of *Lactobacillus* strains on social behavior and their potential to prevent or attenuate features of social impairments.

Antibiotic depletion of mice GM in another neurodevelopmental window, adolescence, was not associated with decreased sociality, but with impaired social memory (Desbonnet et al., 2015). This correlated with reduced mRNA levels of oxytocin and vasopressin in the hypothalamus (Desbonnet et al., 2015). GM also modulate myelination in the prefrontal cortex. Hoban et al. (2016) found abnormal hypermyelinated axons in male germ-free mice. Genes involved in myelination and myelin plasticity were upregulated specifically in the prefrontal cortex, which was paralleled by increased protein levels, and thicker myelin sheaths in the prefrontal cortex of germ-free mice. This coincided with an upregulation of neural activity-induced pathways (Hoban et al., 2016). In male germ-free mice bacterially colonized post weaning (postnatal day 21), none of these genes were differentially regulated, suggesting a dynamic influence of host GM on myelin-related and activity-induced genes. Increased myelin protein abundance, however, could not be reversed (myelin formation in mice occurs around postnatal day 10; Hoban et al., 2016).

Although host genetics can influence GM (Stewart et al., 2005), adult monozygotic twins do not have more similar GM than adult dizygotic twins, and genetically unrelated cohabiting partners have more similar GM than unrelated individuals (Turnbaugh et al., 2009; Song et al., 2013). This emphasizes the importance of the environment, including diet, drinking water, sanitation, hygiene and antibiotics, in shaping the GM after early life events (Martínez et al., 2015). GM differences among societies or communities could reflect particularities in the exposure to such factors (Martínez et al., 2015) while social structure and behavior may determine the flow of microbes among individuals (i.e., horizontal transmission, the transmission of endosymbionts from one individual to another; Song et al., 2013). In this sense, natural selection may have favored GM promoting their transmission via social interactions (Stilling et al., 2014). Many of the currently available studies supporting the hypothesis of an influence of the GM on social behavior have been conducted in germ-free mice. The germ-free mouse model presents the major advantage of in proof-of-principle studies, as well as the possibility of introducing certain microbiota or a defined bacterial consortium at various time points of host development (Luczynski et al., 2016). It is worth noting, however, that the possibility of translating such studies is limited, as no equivalent condition exists in wild mammals or humans. Furthermore, upbringing of germ-free mice may induce irreversible neurodevelopmental deficits, along with a range of other impairments that may limit the suitability of the model for specific scientific queries. Also, studies in a germ-free model do not allow disentangling cause and effect; in non-germ-free laboratory mice, other model organism or wild animals’ changes in behavior and/or in the brain may influence the type of bacteria present in the gut. The alternatives used, i.e., antibiotic treatment and probiotic feeding, as in some of the other studies presented, can be regarded as potentially more relevant with respect to translation than the use of germ-free mice. Further alternatives include fecal transplantation and mouse humanization (Cryan and Dinan, 2012). Nevertheless, all these findings suggest that the GM is critical for the development and modulation of the neurobiological substrate of social behavior, and that specific microbial strains might promote host social behavior. They further suggest that the prenatal and postnatal periods are the most critical neurodevelopmental windows for GMs’ influence on social behavior. Thus, microbial replenishment until adolescence might, to some extent, rescue social deficits based on GM dysbiosis (Table 1). These deficits may include transitory, yet incapacitating, mental states associated with dysbiosis. For instance, symptoms of long-term depression in rodents can be facilitated by the blocking of cellular endocannabinoid uptake (Gerdeman et al., 2002), which can take place during antibiotic-induced dysbiosis. Resembling the lack of motivation found in subjects with bowel disorders, dysbiosis can result in impaired sociality and depression-like symptoms due to neurochemical and functional modifications in the hippocampus; remarkably all could be reversed by administering a probiotic, leading to a normalization of such neurochemical and behavioral modifications (Guida et al., 2018).

**Table 1:** Perturbation of the gut microbiota (GM) can affect social behavior in rodent models.

Model/intervention	Effects on social behavior	Neural correlates	Reference
			
**Prenatal period**			
Maternal immune activation	Offsprings exhibited reduced sociability and reduced preference for social novelty	–	Hsiao et al. (2013)
Maternal high fat diet	Offsprings had fewer social interactions, exhibited reduced sociability and reduced prefeference for social novelty	Reduced oxytocin levels in the hypothalamus	Buffington et al. (2016)
Maternal antibiotic treatment	Offsprings had fewer and shorter social interactions	–	Degroote et al. (2016)
Maternal antibiotic treatment (1 week before delivery to 3 weeks after delivery)	Offsprings exhibited reduced sociability and reduced preference for social novelty. Male offsprings exhibited increased aggressive behavior.	Increased mRNA expression of arginine vasopressin receptor 1b in the frontal cortex	Leclercq et al. (2017)
Maternal antibiotic treatment (1 week before delivery to 3 weeks after delivery)	Prevented decrease in sociability and preference for social novelty in offsprings. No effect on male offsprings aggressivity observed.	Non significant (*p* = 0.1) trend of decreased mRNA expression of arginine vasopressin receptor 1b in the frontal cortex compared to offsprings exposed to antibiotics only.	Leclercq et al. (2017)
**Post natal period**			
Germ-free mice	Increased sociability and increased preference for social novelty	–	Arentsen et al. (2015)
Germ-free mice	Reduced sociability and reduced preference for social novelty	–	Desbonnet et al. (2014)
**Childhood and adolescence**			
Bacterial colonization of socially impaired germ-free mice (at 3 weeks)	Restores sociability but not preference for social novelty, suggesting impaired social memory	–	Desbonnet et al. (2014)
Colonization of socially impaired mice with healthy mice GM (at 4 weeks)	Restores sociability and preference for social novelty	–	Buffington et al. (2016)
Probiotic administration (*Lactobacillus reuteri*) to socially impaired mice (at 4 weeks)	Restores sociability and preference for social novelty	Enhanced oxytocin levels in the hypothalamus	Buffington et al. (2016)
Antibiotic treatment (from 3 weeks onwards)	Normal sociality, reduced social memory	Reduced oxytocin and vasopressin levels in the hypothalamus	Desbonnet et al. (2015)
**Adulthood**			
Colonization of socially impaired mice with healthy mice GM (at 8 weeks)	Fails to restore sociability and preference for social novelty	–	Buffington et al. (2016)

Other findings in rodent models are consistent with knowledge of human brain development. Neurogenesis and neural migration occur prenatally. Synaptogenesis and glycogen synthesis start before birth and continue postnatally, with synaptic density reaching its maximum at 2 years of age (Borre et al., 2014). Furthermore, primates (including humans) show a late and prolonged postnatal neurodevelopment, showing sensitivity to environmental insults (e.g., a chemically-driven developmental impairment) up to the end of adolescence (Borre et al., 2014; Morin et al., 2017), as prefrontal cortex maturation concludes; prefrontal cortex continues its development up until 20 years of age (Marín, 2016).

The ASD is usually characterized by pronounced disturbances of social behavior, and much of the evidence for a connection between GM and social behavior arose from investigations of this malady in human epidemiological studies and from biomedical studies in rodent models. Individuals with ASD present deficits in social communication and interactions, along with stereotypic behavior. Several studies reported that ASD patients have an altered GM composition (Vuong and Hsiao, 2017) and a higher prevalence of inflammatory bowel disease and other gastrointestinal disorders compared to controls (Doshi-Velez et al., 2015). Hsiao et al. (2013) showed that the gastrointestinal symptoms also co-occurred with symptoms in the CNS in an ASD mouse model. They further demonstrated that treatment with the gut bacterium *Bacteroides fragilis* ameliorated GM dysbiosis, corrected gastrointestinal abnormalities, and improved some of the autism-associated behavioral impairments, although deficits in sociability and social preference remained. Desbonnet et al. (2014) demonstrated that germ-free animals exhibited reduced sociability and reduced preference for social novelty. Whereas post-weaning bacterial colonization of germ-free mice reversed social avoidance, it had no effect on social memory impairment (Desbonnet et al., 2014). Findings in a similarly designed study, however, deviated from those of Desbonnet et al. (2014) as germ-free mice exhibited increased sociability and increased preference for social novelty (Arentsen et al., 2015). Interestingly, ASD is typically diagnosed before 2 years of age (Marín, 2016). From then until the end of adolescence, the brain undergoes a process of neurodevelopmental reorganization by synaptic pruning. This makes it vulnerable to environmental deficiencies, including malnutrition (Morin et al., 2017) and GM dysbiosis. Thus, adolescence is a critical period for the onset of several neuropsychiatric disorders including schizophrenia, depression and obsessive-compulsive disorder (Marín, 2016). Another exploratory open label study evaluating an investigative microbial transfer in 18 children with ASD (7–16 years) yielded promising results: the treatment produced significant improvements in both gastro-intestinal and autism-related symptoms, and the GM composition of the ASD children approached that of neurotypical children (Kang et al., 2017).

Perhaps, one of the most significant social impairments may be that of social isolation, often worst endured by the elderly (Weldrick and Grenier, 2018). Evidence suggests, however, that elders who interacted more often with the people in their communities have GM resembling those of younger individuals (Kinross and Nicholson, 2012). As described above, the GM is modified throughout the life cycle. The GM of children lack the complexity found in adult individuals, whereas advancing age has been associated with lower proportions of bifidobacteria and higher proportions of bacteroides (Hopkins et al., 2002). While infant’s GM are represented by *C. leptum and C. coccoides*, those of elderly individuals show higher proportions of *E. coli* and *Bacteroidetes*, suggesting an evolving ratio of Firmicutes to Bacteroidetes across the life cycle (Mariat et al., 2009). Within Firmicutes, significant variations in the butyrate producing genera have been found in elders, including Actinobacteria, Feacalibacterium and Proteobacteria, butyrate-producing taxa providing a major energy source to the intestinal epithelium (O’Toole, 2012). Because of its close relationship with immunity, differences in the composition of GM across different ages may also contribute to the progression of the frailty and poor health observed in old age (Biagi et al., 2010). Therefore, aging may be characterized by a reduction of core GM, accompanied by increments in subdominant and pro-inflammatory species, but also by an enrichment of other health-related bacteria (i.e., *Akkermansia, Bifidobacterium*, or *Christensenellaceae*) that may give extreme elders (>104 years old) some kind of “longevity adaptation” (Biagi et al., 2016).

## Reciprocal Interactions

GM may affect functions of the CNS through different mechanisms, including immune stimulation, enteroendocrine cell activation, microbial production of metabolites with neuroactive properties and vagus nerve stimulation (reviewed in Montiel-Castro et al., 2013). The latter mechanism might be particularly relevant for GM effects on social behavior. The vagus nerve comprises afferent (80%) and efferent fibers and is essential for relaying GM-derived signals to the brain. Many of the effects of the GM on brain function, including some mediated by the *Lactobacillus* species *L. reuteri* and *L. rhamnosus*, depend on vagal activation (Bercik et al., 2011). Importantly, the main central relay for vagal afferent information, the *nucleus tractus solitarii*, projects to the paraventricular hypothalamic nuclei (Affleck et al., 2012) and to the parabrachial nucleus, which further connects to the prefrontal cortex and the amygdala (Hoban et al., 2016), thus providing an easy access for GM-derived signals to behaviorally critical brain areas.

## Social Stressors

Reciprocal interactions between microbiota and its host are the result of a long coevolutionary process (McFall-Ngai et al., 2013). Therefore, we should assume that the resulting symbioses are influenced by the environmental conditions in which the holobiont evolves. Thus, any ecological variables (e.g., climate, food availability, pathogen prevalence; Amato, 2013) affecting the reproductive success and/or the survival of macroorganisms should be considered in the analyses of their relationships with microbial communities. In addition, individuals are subject to stress due to social life within their own groups, e.g., due hierarchy-related interactions (Sapolsky, 2005).

The stress response is regulated by the hypothalamic-pituitary-adrenal (HPA) axis, controlling the “fight or flight” response to acute threats, but also allowing for the inadequate or chronic activation of adrenocortical function, resulting in deleterious effects in terms of health and survival (McEwen, 2007). Recently, the relationship between social stressors and GM has been experimentally established, suggesting that the exposure to a social stressor, either acutely (Galley et al., 2014) or chronically (Bailey et al., 2011), can significantly modify the community structure of the GM. A likely culprit mechanism for this effect is that stress affects the permeability of the gut allowing antigens to cross the epithelium and activating immune responses, which in turn affect the composition of the microbiome (Dinan and Cryan, 2012). Stress may influence GM even before birth, decreasing the genus *Lactobacillus* while augmenting* Oscillibacter*, *Anaerotruncus* and *Peptococus* after mother’s prenatal stress (Golubeva et al., 2015). Not surprisingly, newborn postnatal stress can increase the vulnerability to disease in later life (O’Mahony et al., 2009). Two studies in Rhesus macaques (*Macaca mulatta*) suggest that the integrity of the GM communities and particularly that of the *Lactobacillus* population was decreased due to the disruption of the mother-infant bond, leaving those infants with the highest responses to separation as the most susceptible to bacterial infection. The abundance of *Prevotella* was in turn associated to the stress physiology of the period of peer group-formation in young monkeys (Amaral et al., 2017). Remarkably, Amaral et al. (2017) suggested that the formation of artificial social groups with the weaned infants resulted in the convergence of microbial profiles after 2 weeks, suggesting that social interactions continue to homogenize the GM of different individuals, highlighting the influence of affiliation upon this process (*sensu* Montiel-Castro et al., 2013). We hypothesize that similar weaning effects on GM might be observed in nursery vs. home conditions for human babies.

## Solitary and Social Animals

While laboratory studies are essential for understanding cause and effect relationships, studies in wild animals should be crucial for understanding host-microbe coevolution (Amato, 2013). If we consider individual hosts as patches subject to colonization amongst which microbes can transfer, then meta-community dynamics would suggest that individuals further apart from each other should present more dissimilar microbial communities (Amato, 2013). In contrast, the similarity of microbial taxa will be higher in hosts living in groups with high population densities and frequent interindividual contact (Amato, 2013). This means that we should test whether higher affiliation leads to the homogenization of microbial communities between different individuals, and whether microbial community similarities between individuals serve as an index of the strength of subjects’ social bonds and/or group’s social cohesion (Montiel-Castro et al., 2013).

Meta-community dynamics predict that individuals in habitats distant from each other, geographically isolated or in fragmenting populations should exhibit more distinct microbial communities, with lower diversity and representing local subsets of taxa (Amato, 2013). In this sense, we would expect that solitary individuals, those living in small groups or those with weak social relationships should present more dissimilar microbiota (Ezenwa et al., 2016b). The case of locusts (short-horned grasshoppers) is particularly interesting. On the one hand, three microorganisms (*Pantoea agglomerans*, *Klebsiella pneumoniae pneumoniae*; *Enterobacter cloacae*) inhabiting locusts’ guts produce components of the locust cohesion pheromone involved in their ability to change from its solitary to its swarming gregarious form (Dillon et al., 2002). On the other hand, it has been suggested that the infection by the microsporidian parasite *Paranosema (Nosema) locustae* can inhibit aggregation of locusts (*Locusta migratoria manilensis*) by acidifying the gut and thus suppressing the growth of hindgut bacteria producing aggregation pheromones (Shi et al., 2014). Remarkably, this mechanism is reflected in both a reduced production of serotonin (involved in the initiation of gregariousness, as well as in the suppression of the biosynthesis of the neurotransmitter dopamine maintaining gregariousness: Shi et al., 2014). Nonetheless, this finding raises the exciting question whether the microorganism *Paranosema (Nosema) locustae*, or one with similar capacities, could be present in the gut of patients suffering from psychiatric disorders related to alterations in the production of serotonin or dopamine. This may actually be true across a variety of taxa, since other species of bees, *Apis mellifera* and *Bombus sp*., harbor GM profiles different to those found in solitary species (Engel et al., 2012).

Different mammal societies present social dynamics that can be characterized by variations in terms of party size, party composition and spatial cohesion (Aureli et al., 2008). This allows testing whether such variations are reflected in concurrent modifications between the degree of sociality and the similarities of microbiota across different individuals (*sensu* Montiel-Castro et al., 2013). Evidence suggests that while a more cohesive non-human primate species (*Alouatta pigra*) shows a more homogenous microbial diversity, one showing fluid fission-fusion dynamics has higher microbial dissimilarities between individuals (Amato, 2013). Likewise, in a study on hyenas and microbiota in scent glands, alterations in specific microbial taxa that covaried with volatile fatty acids were different in social vs. solitary hyenas, suggesting relationships between microbiome community composition, odorous signals and social behavior (Theis et al., 2013).

## Social Structure

The social structure of a population includes the nature, quality and patterning of relationships among individual group members (Whitehead, 2008). Group living offers many advantages, such as cooperative foraging, mutual protection, or privileged position for finding a mate; however, it also entails a major disadvantage: increased risk of acquiring communicable diseases by pathogen infection (Altizer et al., 2003). Therefore, how can large groups and high probabilities of microbial transmission coexist in the same populations and/or evolve in parallel, in intensely social species? An important notion is that behavioral responses aid individuals’ physiology, and in particular immune functions, to avoid disease (Pacheco-López and Bermúdez-Rattoni, 2011). Indeed, parasites may impose selective pressures that can drive evolutionary changes in their hosts’ behavior (Møller et al., 1993). Multiple strategies of anti-parasite defense can be observed across many species (Schaller, 2011). Some are reactive, as inhibition (Dantzer and Kelley, 2007) and self-medication (Dantzer and Kelley, 2007), but many animals also prevent infection by activating pre-emptive responses, including aversive emotions, behavior and cognitions, before actual physical interaction with sick individuals (Schaller, 2011). Collectively, these behavioral strategies have been linked to a “social” or “behavioral immune system” (Schaller, 2011). Such strategies could be involved in forming the basis of societal structures and behaviors, including patterns of gregariousness, conspecific-perception, intergroup prejudice and mate preferences (Cremer et al., 2007). The responses inherent to social and behavioral immunity also allow for the transmission of beneficial microbes: duelling positive and negative feedback loops could simultaneously select for behaviors that help hosts acquire beneficial microbes and select against behaviors that increase the transmission of pathogenic microbes (Ezenwa et al., 2016a). Hosts are exposed to endosymbiotic microbes through many behaviors. If microbes benefit host fitness, hosts should behave in ways that promote the acquisition of beneficial microbes, favoring the spread of beneficial microbes across hosts’ populations (Montiel-Castro et al., 2013). Recent evidence suggests that social structure and behavior are important forces shaping the GM composition of individual animals (Koch and Schmid-Hempel, 2011b; Tung et al., 2015; Moeller et al., 2016b; Amato et al., 2017).

Troyer (1984) argued that the advantage provided by the acquisition from conspecifics of mutualistic microbes enhancing the hosts’ digestive abilities might have influenced the evolution of social systems in herbivores. More recent evidence for a role of endosymbiotic microbes in host protection from pathogens prompted Lombardo (2008) to extend Troyer’s hypothesis to non-herbivores and to propose that socially transmitted GM may also protect their hosts from pathogens. Evidence supporting this theory comes from bumblebees (*Bombus terrestris*), which harbor specialized GM that are absent in solitary bee species (Koch and Schmid-Hempel, 2011a). To acquire these specific GM, the bumblebees have to be exposed to feces from nest mates (Koch and Schmid-Hempel, 2011b). This socially transmitted GM protected their host against the widespread and virulent parasite *Crithidia bombi* (Koch and Schmid-Hempel, 2011b).

More recently, Montiel-Castro et al. (2013) reflected on microbial transmission in primates. Primates live sometimes in large groups and tie long-lasting social bonds (Mitani, 2009), which increase their risk of exposure to parasites. Yet, primates can recognize individuals and exert partner-choice (Cheney and Seyfarth, 1982). In this process, they may also use their “behavioral immune system toolbox”; in particular olfaction. Mandrills (*Mandrillus sphinx*) can gauge the parasitic status of group members by sniffing their feces and avoid long grooming sessions with parasitized individuals (Poirotte et al., 2017). In many human cultures, greeting behaviors are common that, while called “kisses”, could be better described as “sniffing” and could serve a similar function (Montiel-Castro et al., 2013). Primate societies are often structured in such a way that the strength of interactions between partners is variable and individuals do not interact with every member of the group, suggesting that information of any kind, including inter-individual microbial exchange, would not be transmitted across the whole social network (Dunbar, 2011). Hence, Montiel-Castro et al. (2013) argued that if the transmission of endosymbionts is beneficial to primate sociality, we should find more intense endosymbiont transmission where social bonds between individuals were stronger. This would allow for the beneficial exchange of mutualistic endosymbionts between individuals, while simultaneously permitting the partner choice mechanisms found in structured societies and limiting the extent of parasite transmission. Social behaviors typically observed between individuals with strong social bonds may allow the horizontal transmission of GM; good examples are social grooming in non-human primates, or human kissing.

Recent studies adopting this concept in non-human primates suggest that the strength of interactions between individuals contributes substantially to shaping the individuals’ GM. Tung et al. (2015) demonstrated that the GM of wild yellow baboons (*Papio cynocephalus)* from two different social groups differed, although both groups exploited adjacent habitats and ate similar diets. Furthermore, within social groups, closer grooming partners had more similar GM, and such relationship extended to the functional similarity of the gut microbiomes (Tung et al., 2015). Socially structured non-human primates consistently harbor *Bifidobacteria*, a group of bacteria that has been linked to beneficial health effects in humans (Tung et al., 2015). For instance, in wild black howler monkeys (*Alouata caraya*), primates that live in small social groups with relatively little social interaction, adult female-female dyads who spent more time in contact or in close proximity had more similar GM than other group members (Amato et al., 2017). Black howler monkey adult females generally interact more with each other than with males, or than males with other males. Interestingly, the effect of social interactions on GM was smaller for black howler monkeys than for baboons (Tung et al., 2015). Together, these observations suggest that social interactions could strongly influence GM composition, particularly for individuals living in large social groups and spending ample time in social interactions (Amato et al., 2017). For example, over the course of a lifetime, chimpanzees acquire most of their gut phylotypes horizontally, i.e., through social interactions, rather than vertically from parent to offspring (Moeller et al., 2016b). Moreover, the GM of wild eastern chimpanzees (*Pan troglodytes schweinfurthii*) from the Kasekala community (Tanzania) were more similar throughout a season during which chimpanzees were more sociable than during a season when they were less sociable, and sociability was positively associated with GM species richness (Moeller et al., 2016b). A crucial property of any society is the proportion of time that its members spend in the vicinity of, or in physical contact with, conspecifics, often used as a measure of the intensity of their social relationships (Wilson, 2000). Indeed, interchange of GM may occur via indirect transmission: where susceptible hosts are exposed to microbial life via an environmental feature (Cortez and Weitz, 2013). For example, co-habiting, genetically unrelated partners harbor more similar gut bacterial communities than individuals living in different households (Yatsunenko et al., 2012; Song et al., 2013; Mosites et al., 2017). Presumably, this effect is largely due to the consumption of similar foods, as members of a household likely eat similar diets. Another study, investigating bacterial communities from the skin of household members and from surfaces in their houses, demonstrated that houses harbor a specific microbial fingerprint shaped by the household members (Lax et al., 2014). Members of a household had more similar skin microbiota than those not sharing a home, and in one of the households examined, the two occupants that formed a couple shared more of their skin microbiota with each other than with a third housemate (Lax et al., 2014). Interestingly, kissing homogenizes the oral microbiota of human couples (Kort et al., 2014). It remains unknown, however, whether some of the microbes exchanged by kissing might also find a niche in the gut, contributing to the fact that cohabiting partners have more similar GM that unrelated individuals (Song et al., 2013).

This allows considering a possible association between the direct transmission of microbial life (i.e., via physical contact between infected and susceptible conspecifics; Cortez and Weitz, 2013) and the intensity of social relationships. In this sense, sexual interactions represent both direct means of microbial transmission and a crucial aspect of social life. For instance, given their reproductive anatomy, studies in reptiles and birds can provide interesting angles on whether GM are transmissible via sexual interactions (White et al., 2010). White et al. (2011) found that the cloacal bacterial communities of polyandrous female lizards (*Zootoca vivipara*) were significantly higher than in monandrous females, suggesting that a larger number of sexual partners increased bacterial diversity in females’ cloaca. Likewise, White et al. (2010) experimentally demonstrated microbial transmission via sexual interactions in kitiwakes (*Rissa tridactyla*) by first allowing and then impeding insemination, finding that: (a) cloacal diversity decreased; and (b) the microbial communities of mates became more dissimilar after the experimental blocking took place (White et al., 2010). In another bird example, male barn swallows (*Hirundo rustica erythrogaster*) interacting primarily with males had a lower microbial diversity than females interacting with various males (Levin et al., 2016). A series of studies focused on microbial diversity across sexes and a variety of species may indicate that the sex that interacted with the widest diversity of social or ecological niches was also the one registering the largest microbial diversity (reptiles: (*Zootoca vivipara*) White et al., 2011; birds: (*Rissa tridactyla*) White et al., 2010; (*Hirundo rustica erythrogaster*) Levin et al., 2016; mammals: (*Suricata suricatta*) Leclaire et al., 2014; (*Alouatta pigra*) Amato et al., 2017). Nonetheless, there is still a big gap in the animal taxa in which a GM association with social behavior has been investigated. Research models such as those used in the emerging field of wild immunology (Babayan et al., 2011) might prove useful to investigate such questions and bridge the gap between laboratory studies in animal models and ecological studies of wild animals. Wild animals, notably social animals that serve as reservoirs for human pathogens, might also provide interesting topics for further research. Such studies may help to understand the nature of GM’s role in infectious disease ecology and evolution. For instance, the common vampire bat (*Desmodus rotudus*), a species that maintains the rabies virus in the wilderness (Benavides et al., 2016), could be particularly interesting in this context, as it lives in large groups, exhibits complex and structured societies, practices social grooming and shares food by regurgitation (Carter and Leffer, 2015).

In humans, the GM are increasingly recognized as important modulators of autoimmune diseases (Kosiewicz et al., 2011) that have recently increased dramatically, particularly in high-income countries (Rook, 2013). The “hygiene hypothesis” explains this increase by a reduced exposure to “old friends” (microbiota of humans, animals, viruses, or parasites from the environment): organisms with which humans coevolved, and whose inputs are essential for the immune system to develop normally (Rook, 2013). Interestingly, two bacterial genera that are linked to social behavior, *Lactobacillus* (which modulated social behavior in mouse models: Buffington et al., 2016) and *Bifidobacterium* (which were socially structured in baboons; Tung et al., 2015), have been shown to be protective in several allergic and autoimmune diseases, including colitis, type-1 diabetes and experimental autoimmune encephalomyelitis (reviewed in Kosiewicz et al., 2011). Hence, whether socially transmitted gut bacteria might benefit their host by contributing to pathogen defense and preventing autoimmunity is an interesting open question. Industrialized societies appear to have lower bacterial diversity within individuals, and less similar GM between individuals than non-industrialized societies (Schnorr et al., 2014). In this context, we should consider whether societal characteristics might limit microbial transmission. Relative to other countries, average household size is generally small in high-income countries (United Nations, 2013), with life styles associated to a shrinking social network (McPherson et al., 2006). Else, access to increased sanitation and hygiene may influence the extent or type of microbes shared. Yet, while differences in social GM transmission may somehow contribute to the increased burden of immunopathological diseases in high-income countries, they are certainly not its primary cause. Alterations in GM composition resulting from changes in diet, antibiotic overuse, reduced exposure to diverse microbes from the environment, or elimination of constitutive partners such as nematodes, may likely contribute substantially to this trend (Okada et al., 2010). In this case, restrained social transmission of GM might just make things worse, notably by promoting the loss of valuable biological assets: endosymbionts.

## Conclusion

Evidence emerging from biomedical and ecological studies suggests that GM plays an important role in shaping host sociality and that, vice versa, social organization and behavior influence the GM associated with individuals. The two processes appear to be linked, as microbes acquired through horizontal transmission by mothers can be transmitted vertically to the next generation. Gestation and infancy appear to be critical periods for the GM to influence the infant’s brain development, and may be critical in determining its future degree of sociability (Figure 1).

These findings reinforce earlier hypotheses proposing that gregariousness, social structures, and social behaviors might in part have evolved because they enhance or fine-tune the beneficial transmission of endosymbionts (Troyer, 1984; Lombardo, 2008; Montiel-Castro et al., 2013). Simultaneously, some co-evolving gut bacteria might have gotten involved in modulating their host’s sociality because this would increase their own transmission between hosts. The mutual dependency between macroorganisms and symbionts, has been highlighted here by a focus on the reciprocal interactions between microorganisms and social processes at the macroorganism level. This evidence suggests that the holobiont may in fact be a “unit of selection”, understood both as a “replicator” or an “interactor” (Lloyd, 2017). While group living increases the risk of exposure to pathogens, the social transmission of beneficial GM may compensate for this risk if it increases the hosts resistance to infectious agents (Ezenwa et al., 2016b). Findings in insects directly link socially transmitted GM to pathogen resilience (Koch and Schmid-Hempel, 2011b); it will be interesting to see whether this generalizes to mammals.

Beyond shedding light on the ecology and evolution of species, such findings may have major implications for human health. The fact that specific GM may shape social behavior could eventually translate into GM-based therapies for mental disorders associated with social deficits, i.e., psychobiotics (Vétizou et al., 2015). Also, closer examination of the long-term consequences of GM establishment during early life and how maternal diet, mode of delivery and feeding, and antibiotic treatments influence this process might optimize recommendations and nutritional interventions to promote a healthy brain development (Goyal et al., 2015). The sharing of GM through group living and social interactions may be relevant for human health as well, as group promoted or socially transmitted GM may support a healthy brain development and determine individual disease susceptibility. Industrialized societies have lower bacterial diversity within individuals, and less similar GM between individuals than non-industrialized societies, which may be mirrored in differences in the susceptibility to infectious and autoimmune diseases. This raises the question of to what extent differences in social structures and behaviors limit the horizontal transmission of potentially protective GM and, hence, contribute to these observations.

Overall, it appears that the microbes, in particular GM, and host social behavior have coevolved to become virtually inseparable. The GM appear to shape the social behavior and structure of their hosts, and depend on them for transmission. In turn, GM offer benefits by protecting from several diseases. Further investigation of these fascinating reciprocal interactions may open avenues for the treatment of neurological disorders or the management of health and diseases with an evolutionary perspective. Moreover, investigating these reciprocal interactions may advance our understanding of sociality, from social cognition to the basis of society structures.

## Author Contributions

EM and GP-L initiated this work as part of the ETH Zurich course 701-1701-00L “Human Health, Nutrition, and Environment: Term Paper” during the autumn semester 2016. All authors listed, have made substantial, direct and intellectual contribution to the work, and approved it for publication. In particular: EM, AM-C and GP-L performed the literature review. EM, AM-C, WL and GP-L wrote the manuscript. EM designed the figures.

## Conflict of Interest Statement

The authors declare that the research was conducted in the absence of any commercial or financial relationships that could be construed as a potential conflict of interest.
